# Expressions and clinical significance of CCN5 and E-cadherin in primary and recurrent lesions of breast cancer

**DOI:** 10.3389/fgene.2024.1404515

**Published:** 2024-07-31

**Authors:** Guofeng Zhou, Xingxing Gui, Wei Qu, Xiujuan Zhang

**Affiliations:** ^1^ Department of Pathology, The Third Hospital of Nanchang, Nanchang, China; ^2^ Department of Ultrasound, The Third People’s Hospital of Ji’an City, Ji’an, China

**Keywords:** breast cancer, primary lesion, recurrent lesion, CCN5, E-cadherin, metastases

## Abstract

**Background:**

Breast cancer recurrence and lymph node metastasis significantly impact patient outcomes. Understanding the molecular mechanisms behind these processes is crucial for developing effective treatments. CCN5 and E-cadherin are proteins involved in cell adhesion and epithelial-mesenchymal transition (EMT), playing roles in breast cancer progression.

**Objective:**

This study aimed to analyze the expression levels and clinical significance of CCN5 and E-cadherin in primary and recurrent breast cancer lesions.

**Methods:**

Immunohistochemical staining using the SP method was performed to detect CCN5 and E-cadherin expression levels in 28 normal breast tissue samples, 52 primary breast cancer lesions, and paired recurrent chest wall lesions. The expression levels of these proteins were compared across different tissue types and correlated with lymph node metastasis.

**Results:**

CCN5 and E-cadherin expression levels significantly differed among normal breast tissues, primary breast cancer lesions, and recurrent lesions (Χ^2^ = 18.934 and Χ^2^ = 14.516, *p* < 0.05). Primary breast cancer lesions exhibited higher CCN5 and E-cadherin expression levels compared with recurrent lesions and normal tissues, although these differences were not statistically significant. Patients without lymph node metastases exhibited significantly higher expression levels of CCN5 and E-cadherin compared with those with lymph node metastases (Χ^2^ = 9.775, Χ^2^ = 9.1479, *p* < 0.05). A positive correlation between CCN5 and E-cadherin expression levels was found in breast cancer tissues (r = 0.398, *p* < 0.001).

**Conclusion:**

CCN5 and E-cadherin were expressed at lower levels in recurrent breast cancer tissues and those with lymph node metastases, indicating their potential roles in breast cancer recurrence and metastasis. These findings suggest that CCN5 and E-cadherin might work synergistically to influence breast cancer progression.

## Introduction

Breast cancer represents a significant public health challenge, with its incidence steadily rising since the 1970s, making it a major concern worldwide (Harbeck and Gnant, 2017). Lymph node metastases and local recurrence stand as pivotal determinants of breast cancer prognosis, exerting substantial influence on patient outcomes. Understanding the molecular mechanisms underlying tumor growth, invasion, and metastasis is crucial for developing effective therapeutic strategies. The intricate processes governing cell mitosis, intercellular adhesion, apoptotic induction, and extracellular matrix generation play notable roles in cancer progression (Shah and Gallick, 2007; Barreto et al., 2016). Among the proteins implicated in these processes, CCN5 and E-cadherin have noticeably attracted oncologists’ attention in breast cancer research.

CCN5, also known as WISP-2, belongs to the CCN family of proteins and participates in diverse cellular functions, including angiogenesis, cell proliferation, differentiation, apoptosis, and tumor formation (Perbal and Perbal, 2016; Hu et al., 2019). Emerging evidence suggests that CCN5 may act as a tumor suppressor by inhibiting epithelial-mesenchymal transition (EMT), a critical step in cancer metastasis. Notably, CCN5 expression level has been reported to be associated with delayed progression of ductal carcinoma *in situ* to invasive carcinoma (Kleer, 2016; Zhang and Han, 2016).

In contrast, E-cadherin, a calcium-dependent transmembrane glycoprotein encoded by the CDH1 gene, serves as a pivotal regulator of cell-cell adhesion and plays a crucial role in maintaining tissue integrity (Banerjee and Banerjee, 2012). Loss or downregulation of E-cadherin expression level is a hallmark of EMT, facilitating tumor invasion and metastasis in various cancer types, including breast cancer. However, the exact mechanisms linking E-cadherin to breast cancer progression remain under investigation (Banerjee et al., 2008; SANDIPTO et al., 2017).

Given the complex interaction among CCN5, E-cadherin, and breast cancer progression, the present study aimed to elucidate their expression levels and clinical significance in primary and recurrent breast cancer lesions. By examining their roles in tumor recurrence and lymph node metastasis, valuable insights were provided into the underlying mechanisms driving breast cancer progression.

## Materials and methods

### Sample sources

Surgical specimens were obtained from patients undergoing modified radical surgery for breast cancer. Normal breast tissue samples were collected from individuals without a history of breast cancer undergoing breast surgery for non-neoplastic conditions. Biopsies and crude needle punctures were performed to confirm recurrent lesions. All samples were processed and embedded in paraffin for histological analysis. This study was conducted in accordance with the provisions of the Declaration of Helsinki. It was approved by the Ethics Committee of The Third Hospital of Nanchang (Nanchang, China; Approval No. KY2021074). All participants were informed and they signed a fully-informed consent form.

### Main reagents

CC5 protein was detected using concentrated anti-CCN5 rabbit polyclonal antibody (Ab38317), which was purchased from Abcam (the United States), with a working dilution of 1:100. Anti-E-cadherin monoclonal antibody (ready-to-use working solution), anti-E-cadherin secondary antibody, and DAB reagent were purchased from Fuzhou Maixin Biological Development Co., Ltd. Other reagents included xylene, gradient ethanol (100%, 90%, 85%), 3%H_2_O_2_, PBS, and hematoxylin.

## Methods

To obtain samples of normal breast tissue for comparison, patients who underwent diagnostic procedures for suspected breast pathology were included in the study. These patients did not present with clinically suspicious lesions upon examination, and subsequent histopathological analysis confirmed the absence of any pathological abnormalities in the sampled tissue. Ethical considerations were strictly adhered to throughout the study, and all participants provided informed consent prior to sample collection. All of the samples were fixed in 10% formalin, dehydrated conventionally, embedded in paraffin, and subjected to HE staining. The immunohistochemical SP method has been widely used for detecting protein expression levels in various tissues (Shah and Gallick, 2007). PBS in place of primary antibody was used as the negative control. Positive breast cancer sections were used as positive controls. The immunohistochemical detection consisted of the following steps (Harbeck and Gnant, 2017): The sections were baked in an oven at 60°C overnight (Barreto et al., 2016); the sections were dewaxed and hydrated (Shah and Gallick, 2007); heating antigen retrieval was carried out using an autoclave and citric acid buffer (Hu et al., 2019); the sections were incubated with deionized water with 3% H_2_O_2_ for 10 min to block endogenous peroxidase (Perbal and Perbal, 2016); primary antibody was added dropwise to incubate the sections at 4°C in a fridge overnight (Zhang and Han, 2016); secondary antibody was added dropwise to incubate the sections at room temperature for 18 min (Kleer, 2016); DAB reagent was added for color development (Banerjee and Banerjee, 2012); the sections were counterstained, dehydrated, transparentized, and sealed with neutral balsam. The method for calculating staining scores based on staining intensity and the percentage of positive cells has been previously described (Kleer, 2016).

### Result interpretation

Positive CCN5 expression was defined if brownish yellow particles were observed in the cytoplasm. Positive E-cadherin expression was defined if yellow or brownish yellow particles were observed in the cell membrane or cytoplasm near the cell membrane. The staining results were interpreted using the double-blind method based on two indicators, namely, staining intensity and percentage of positive cells (Harbeck and Gnant, 2017): No positive staining was scored 0 point, light yellow one point, brownish yellow two points, and brown three points (Barreto et al., 2016). Ten high-power fields were randomly selected, and the percentage of positive cells in every 200 cells was estimated for each field. An average was taken for the 10 fields. The scoring scale utilized in this study to assess CCN5 and E-cadherin expression levels was adapted from previous research (Kleer, 2016). This scoring system assigns points based on staining intensity and the percentage of positive cells, with a total score ≤3 considered negative and a total score >3 considered positive. The utilization of a standardized scoring scale in this study allowed for the objective assessment of CCN5 and E-cadherin expression levels in breast cancer tissues (Kleer, 2016).

### Data collection

Patient demographics, including age, locality, and other relevant variables, were collected from medical records. Clinical data such as tumor stage, hormone receptor status (estrogen receptor, progesterone receptor), and HER2/neu status were also recorded.

### Statistical analysis

SPSS 19.0 software (IBM Corp., Armonk, NY, USA) was utilized to carry out statistical analysis. Nonparametric tests, specifically the Chi-square test, were employed to compare the expression levels of CCN5 and E-cadherin in different tissues. The positive expression rates of CCN5 and E-cadherin in groups with and without lymph node metastases were also compared using the Chi-square test. To assess the correlation between CCN5 and E-cadherin expression levels in breast tissues, Spearman’s rank correlation coefficient was used. All statistical tests were two-sided, and *p* < 0.05 was considered statistically significant.

## Results

Female patients with breast cancer who underwent surgical treatment at our hospital from 1 January 2019, to 31 December 2022, were recruited. The participants ranged in age from 25 to 80 years, with a median age of 40 years.

Statistical comparisons of CCN5 and E-cadherin expression levels among the three groups (normal tissues, primary lesions, and recurrent lesions) were conducted using appropriate non-parametric tests due to the unequal sample sizes. Specifically, the Kruskal–Wallis test was employed to assess differences between groups, followed by Dunn’s *post hoc* test for pairwise comparisons if significant differences were detected.

### CCN5 and E-cadherin expression levels in different types of breast tissues

**FIGURE 1:**
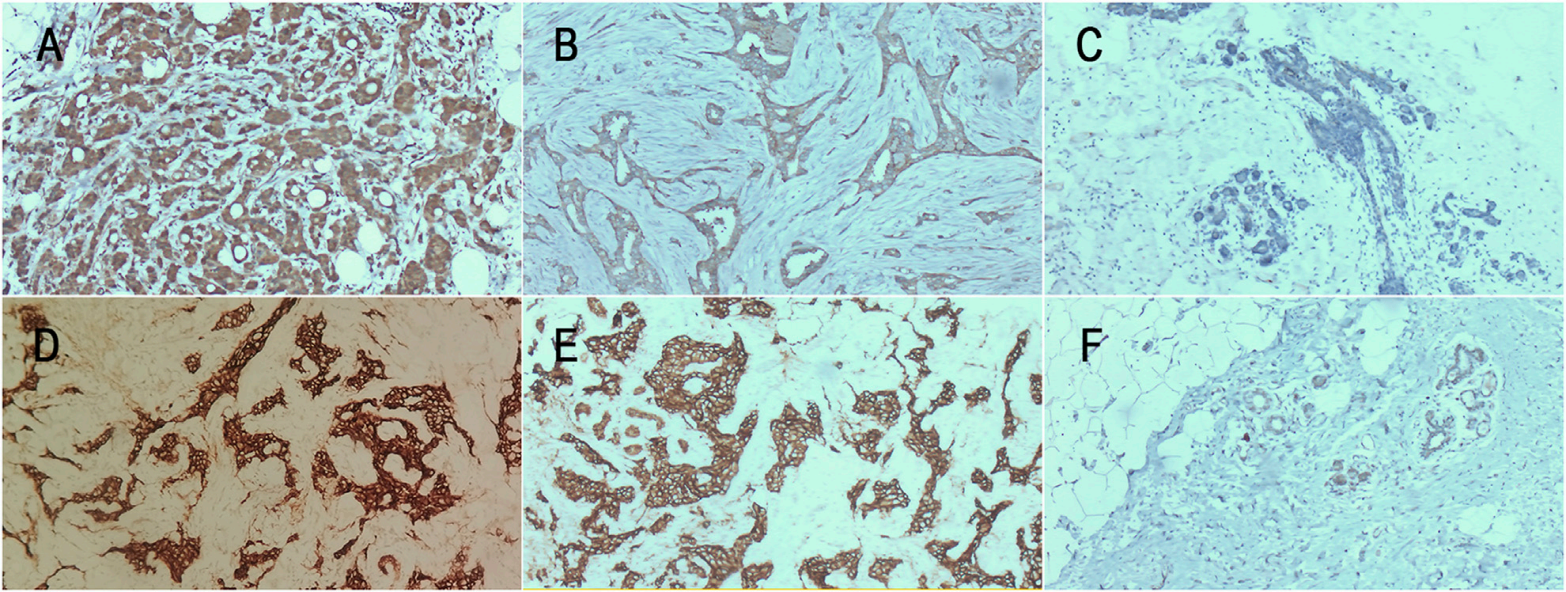
CCN5 and E-cadherin expression levels in each group. **(A)** CCN5 expression level in primary lesion (×200); **(B)** CCN5 expression level in recurrent lesion (×200); **(C)** CCN5 expression level in normal tissue (×100); **(D)** E-cadherin expression level in primary lesion (×100); **(E)** E-cadherin expression level in recurrent lesion (×200); **(F)** E-cadherin expression level in normal tissue (×40).

Table 1 presents the expressions of CCN5 and E-cadherin in normal breast tissues, primary lesions, and recurrent lesions. CCN5 was positively expressed in four out of 28 normal tissues (14.29%), 33 out of 52 primary lesions (63.46%), and 21 out of 52 recurrent lesions (40.38%). E-cadherin was positively expressed in six out of 28 normal tissues (17.86%), 34 out of 52 primary lesions (65.38%), and 23 out of 52 recurrent lesions (44.23%). The Chi-square test indicated significant differences in the expression of both CCN5 (Χ^2^ = 18.934, *p* = 0.000) and E-cadherin (Χ^2^ = 14.516, *p* = 0.001) across the different tissue types (Figure 1).

**TABLE 1:** CCN5 and E-cadherin expression levels in three different types of breast tissues.

Type of breast tissue	Number of cases	CCN5 expression level (n, %)	E-cadherin expression level (n, %)
Normal tissue	28	4 (14.29)	6 (21.43)
Primary lesion	52	33 (63.46)	34 (65.38)
Recurrent lesion	52	21 (40.38)	23 (44.23)
X^2^		18.934	14.516
P		<0.001	<0.001

Note: Values in parentheses represent the number of cases and the percentage of cases expressing CCN5 and E-cadherin within each group of breast tissues. Χ^2^ represents the chi-square statistic for comparing expressions among the three groups.

### CCN5 and E-cadherin expression levels in breast cancer patients with and without lymph node metastases

As shown in Table 2, among patients with lymph node metastases, six out of 19 (31.58%) expressed CCN5 positively, compared to 25 out of 33 (75.76%) in patients without lymph node metastases. Similarly, seven out of 19 patients (36.84%) with lymph node metastases expressed E-cadherin positively, whereas 26 out of 33 patients (78.79%) without lymph node metastases showed positive expression. The chi-square test revealed significant differences in the expression of CCN5 (Χ^2^ = 9.775, *p* = 0.002) and E-cadherin (Χ^2^ = 9.149, *p* = 0.002) between these groups.

**TABLE 2:** CCN5 and E-cadherin expression levels in breast cancer patients with and without lymph node metastases.

Type	Total number of cases	CCN5 positive (%)	E-cadherin positive (%)
Group with lymph node metastasis	19	6 (31.58)	7 (36.84)
Group without lymph node metastasis	33	25 (75.76)	26 (78.79)
*p*-value		0.002	0.002

### Correlation between CCN5 and E-cadherin expression levels in primary breast cancer

Table 3 illustrates the correlation between CCN5 and E-cadherin expression levels in primary breast cancer tissues. Among the tissues, 45 cases exhibited positive expression levels of both CCN5 and E-cadherin, while 20 cases were positive for CCN5, while negative for E-cadherin. Additionally, 11 cases were negative for CCN5 expression level, while positive for E-cadherin expression level, and 28 cases were negative for expression levels of both markers. The statistical analysis demonstrated a significant correlation between CCN5 and E-cadherin expression levels (Spearman’s rank correlation coefficient: r = 0.398, *p* = 0.01).

**TABLE 3:** Correlation between CCN5 and E-cadherin expression levels in primary breast cancer.

CCN5	E-cadherin	
	Positive	Negative
Positive	45	20
Negative	11	28

Note: Pearson correlation analysis was conducted to evaluate the relationship between CCN5 and E-cadherin expression levels in primary breast cancer tissue samples.

Pearson Correlation Coefficient (r) = 0.398.

*p*-value = <0.01.

## Discussion

### Significance of CCN5 expression in breast cancer

CCN5 (also known as WISP-2) is a member of the CCN family and involved in angiogenesis, cell proliferation, differentiation, apoptosis, and tumor formation. CCN5 is considered to play important roles in regulating the proliferative activity, motility, invasion and adhesion of cells (Perbal and Perbal, 2016). Epithelial-mesenchymal transition (EMT) is an important process during the development of many cancers, including ductal breast cancer (Zhang and Han, 2016). EMT is characterized by the loss of characteristics of epithelial cells, including cell polarity, intercellular adhesion, and absence of E-cadherin expression, along with the expression of the mesenchymal marker vimentin (Shah and Gallick, 2007). CCN5 is an important tumor suppressor gene that prevents the occurrence and development of breast cancer by inhibiting EMT (Banerjee and Banerjee, 2012; Kleer, 2016). Sandipto S et al. found that CCN5 expression delayed the development of ductal carcinoma *in situ* into invasive carcinoma (SANDIPTO et al., 2017). We found that the CCN5 expression in primary breast cancer was higher than that in recurrent breast cancer and normal breast tissues. Besides, the CCN5 expression in the group without lymph node metastases was higher than that in the group with lymph node metastases. Our results agreed with those from foreign studies (Banerjee et al., 2008). The above results indicated that high CCN5 expression inhibited the occurrence and development of breast cancer and lymph node metastases. Another implication was that CCN5 inhibited EMT to reduce the migration of breast cancer cells.

### Clinical significance of E-cadherin expression in breast cancer

E-cadherin is a member of the cadherin family. As a calcium-dependent transmembrane glycoprotein, E-cadherin is mainly expressed in epithelial cells and encoded by the cadherin1 gene (CDH1) localized to chromosome 16q22.1. E-cadherin is an important transmembrane adhesion molecule in epithelial cells, and it is indispensable for forming adherens junctions between mature cells. By interacting with catenin, E-cadherin regulates cell differentiation, proliferation, apoptosis, and signal transduction (Shao et al., 2018). EMT is a process whereby epithelial cells lose their polarity and adhesive properties and acquire the invasive and metastatic characteristics of mesenchymal stem cells. EMT is a key step in tumor cell metastasis (Das et al., 2019). Lowly expressed E-cadherin is an important marker of EMT, and it is involved in tumor cell invasion, the characteristics of stem cells, and drug resistance. Low E-cadherin expression can activate a variety of carcinogenic signaling pathways, such as promoting small GTPases, Ras-related C3 botulinum toxin substrate 1, and mitogen-activated protein kinase (Memni et al., 2016). Causes of low E-cadherin expression or absence of E-cadherin expression include loss-of-heterozygosity of the CDH1 gene, epigenetic regulation, promoter hypermethylation, inhibition of E-cadherin hydrolysis by RNA protein transcription, and E-cadherin switch. In addition, abnormal expression of E-cadherin causes interference to intercellular adhesion, thereby promoting cell migration and resulting in metastasis of primary tumors (Caldeira et al., 2006). A study by NA Tae-Young (Na et al., 2020) showed that promoting the adhesion activity of E-cadherin on tumor cell surface inhibited tumor cell migration and metastasis. Their finding suggests that E-cadherin is a tumor suppressor gene, which is not only related to the expression on the tumor cell membrane, but also to the adhesion activity of E-cadherin. Therefore, downregulating E-cadherin can cause a decrease in the adhesive property of epithelial tumor cells, which further promotes the invasion and metastasis of tumor cells. In the present study, we found that the E-cadherin expression in primary breast cancer was higher than that in recurrent breast cancer and normal breast tissues. Besides, the E-cadherin expression in the group without lymph node metastases was higher than that in the group with lymph node metastases. This result implies that E-cadherin plays crucial roles in the recurrence and metastasis of breast cancer.

### Correlations between CCN5 and E-cadherin expressions in breast cancer

The correlation analysis revealed a significant but weak positive correlation between CCN5 and E-cadherin expression levels in breast tissues (r = 0.398, *p* < 0.01), indicating a modest association between these two proteins. While statistically significant, the weak strength of the correlation suggests that other factors may influence the expression patterns of CCN5 and E-cadherin in breast cancer progression. In addition to the findings of the present study, it is important to consider existing literature on the prognostic significance of CCN5 and E-cadherin in breast cancer. Several studies have investigated these markers (Das et al., 2017; Shao et al., 2018; Na et al., 2020), yet the results have been inconsistent. For instance, it was previously reported that high CCN5 expression level was associated with less frequent disease progression and suppressed invasive phenotypes of tumor cells, indicating a potential role as a tumor suppressor (Kleer, 2016). Conversely, other studies have reported no significant correlation between CCN5 expression and clinical outcomes (Sandipto et al., 2017). Similarly, the role of E-cadherin in breast cancer prognosis has been widely studied (Das et al., 2017). While some research suggests that low E-cadherin expression level is linked to increased tumor invasiveness and poor prognosis, no clear association was definitely reported (Na et al., 2020). These discordant results may be due to variations in study design, sample sizes, patient populations, and methodologies used to assess protein expression. Additionally, differences in breast cancer subtypes and the molecular heterogeneity of the disease could contribute to the variability in findings. Acknowledging these inconsistencies, the present study provided further evidence supporting the potential prognostic value of CCN5 and E-cadherin in breast cancer. Both CCN5 and E-cadherin negatively regulated breast cancer via the EMT mechanism (Das et al., 2017). However, we still know little about the mechanism underlying the correlations between CCN5 and E-cadherin expressions in breast cancer. Chai et al. (2019) found that CCN5 expressed in esophageal cancer inhibited invasion and metastasis by downregulating Sul and upregulating E-cadherin. Our results suggested that CCN5 and E-cadherin were simultaneously downregulated in breast cancer, and that the expressions of the two proteins were positively correlated. We can infer that CCN5 and E-cadherin work synergistically in the recurrence and metastasis of breast cancer, though the specific mechanism remains to be further revealed. However, further research with larger, more homogenous cohorts and standardized assessment methods is needed to clarify the prognostic significance of these markers.

One limitation of this study is the absence of detailed clinical data, such as clinical stage and biological profiles (HER2/neu, estrogen receptor, and progesterone receptor statuses), which could provide further insights into the influence of these factors on the presence or absence of CCN5 and E-cadherin expression levels.

The findings of the present study suggested that CCN5 and E-cadherin expression levels could serve as valuable biomarkers in breast cancer, with potential benefits for clinical practice. The differential expression levels of these proteins in primary versus recurrent breast cancer lesions and in cases with or without lymph node metastases highlight their possible roles in monitoring disease progression and recurrence. Specifically, the higher expression levels of CCN5 and E-cadherin in primary lesions and their lower expression levels in recurrent lesions and tissues with lymph node metastases may assist clinicians to identify patients who are at the higher risk of recurrence and metastasis. Clinically, assessing the expression levels of CCN5 and E-cadherin could improve personalized treatment plans. For instance, patients with low expression levels of these markers may benefit from more aggressive treatment strategies and closer follow-up to manage the higher risk of recurrence and metastasis. Additionally, therapies aimed at upregulating CCN5 and E-cadherin expression levels might be explored as potential approaches to inhibit tumor progression and metastasis, providing new insights for targeted therapies. Moreover, the positive correlation between CCN5 and E-cadherin expression levels suggests that combined detection of these markers could enhance the accuracy of prognostic assessments. By integrating these biomarkers into routine diagnostic and prognostic protocols, clinicians could make more informed decisions, ultimately improving patient outcomes.

In conclusion, the expression levels of CCN5 and E-cadherin in primary and recurrent breast cancer lesions, as well as in normal breast tissues were assessed, revealing their potential roles in breast cancer progression and metastasis. The findings indicated significant differences in CCN5 and E-cadherin expression levels across different types of breast tissues, with primary lesions exhibiting higher expression levels compared to recurrent lesions and normal tissues. Moreover, patients without lymph node metastases exhibited significantly higher expression levels of both CCN5 and E-cadherin compared with those with lymph node metastases, suggesting a potential association between these proteins and metastatic spread. Importantly, a positive correlation was identified between CCN5 and E-cadherin expression levels in primary breast cancer tissues, indicating a potential synergistic effect in breast cancer recurrence and lymph node metastases. These results highlight the clinical significance of assessing CCN5 and E-cadherin expression levels as potential biomarkers for predicting breast cancer recurrence and metastasis. Overall, the findings contribute to the growing body of evidence on the roles of CCN5 and E-cadherin in breast cancer progression and provide valuable insights for future research and clinical practice.

## Data Availability

The original contributions presented in the study are included in the article/Supplementary Material, further inquiries can be directed to the corresponding author.
